# Relationship between optimism and quality of life in patients with two chronic rheumatic diseases: axial spondyloarthritis and chronic low back pain: a cross sectional study of 288 patients

**DOI:** 10.1186/s12955-015-0268-7

**Published:** 2015-06-10

**Authors:** Sarah Kreis, Anna Molto, Florian Bailly, Sabrina Dadoun, Stéphanie Fabre, Christopher Rein, Christophe Hudry, Franck Zenasni, Sylvie Rozenberg, Edouard Pertuiset, Bruno Fautrel, Laure Gossec

**Affiliations:** Institute of Psychology, Laboratoire Adaptations Travail – Individu, University Paris Descartes, Sorbonne-Paris cité, Paris, France; Sorbonne Universités, UPMC Univ Paris 06, GRC-08, Institut Pierre Louis d’Epidémiologie et de Santé Publique, Paris, France; Rheumatology Department, Pitié-Salpêtrière Hospital, AP-HP, Paris, France; Rheumatology Department, Cochin Hospital, AP-HP. INSERM (U1153): Clinical Epidemiology and Biostatistics, PRES Sorbonne Paris-Cité, Paris Descartes University, Paris, France; Pontoise University Hospital, Pontoise, France; CHU Pitié Salpêtrière, Service de Rhumatologie, Pavillon Delessert, 47-83 bd de l’hôpital, 75013 Paris, France

**Keywords:** Quality of life, Axial spondyloarthritis, Chronic low back pain, Optimism, Personality traits

## Abstract

**Background:**

Axial Spondyloarthritis (AxSpA) and chronic low back pain are rheumatic diseases that impact patients’ health-related quality of life (HRQoL). In other chronic conditions, HRQoL was positively associated with dispositional optimism, a personality trait. The objective was to explore the relationship between optimism and HRQoL in these two diseases.

**Method:**

A cross-sectional study was performed in 2 tertiary care hospitals and 2 private practices in France. Patients had definite AxSpA or chronic low back pain according to the rheumatologist. A generic HRQoL questionnaire (Short Form, SF-12) with physical and mental composite scores (PCS and MCS respectively) and an optimism questionnaire (the Life Orientation Test-revised, LOT-R) were collected. Analyses included non-parametric correlations and multiple regression analyses to study the effect of optimism on PCS and MCS.

**Results:**

In all, 288 (199 AxSpA and 89 low back pain) patients were included: mean age, 47.3 ± 11.9 years, 48.6 % were males. Pain levels (0–10) were 4.5 ± 2.4 and 4.3 ± 2.4 in AxSpA and LOW BACK PAIN patients, respectively. HRQoL was similarly altered in both diseases, for both physical and mental composite scores (mean PCS: 43.7 ± 8.2 vs. 41.9 ± 7.1; mean MCS: 45.9 ± 7.8 vs. 46.7 ± 8.1 for AxSpA and low back pain respectively). Optimism was moderate and similar in both populations. Optimism was positively correlated to MCS in both diseases (rho = 0.54 and 0.58, respectively, both *p* <0.01) and these relations persisted in multivariate analyses (beta = 1.03 and 1.40, both *p* <0.0001).

**Conclusions:**

Optimism was similar in these 2 chronic diseases and was an explanatory factor of the mental component of HRQoL, but not physical HRQoL. Physical HRQoL may reflect more the disease process than character traits.

**Electronic supplementary material:**

The online version of this article (doi:10.1186/s12955-015-0268-7) contains supplementary material, which is available to authorized users.

## Introduction

Axial Spondyloarthritis (AxSpA) is a chronic inflammatory rheumatic disease affecting primarily the axial skeleton and sacroiliac joints. AxSpA is a painful and potentially disabling condition, typically diagnosed in early adulthood and its usual characteristics are pain, stiffness due to inflammation and impaired physical function [1]. Non-specific low back pain is the most common spinal disorder and is defined as low back pain not related to a known specific pathology such as a tumour, infection, osteoporosis, fracture or inflammatory disorder. Low back pain is defined as chronic when the pain persists for more than 3 months and it represents between 11–12 % of all low back pain cases [2]. Chronic low back pain also leads to pain and impaired physical function. These axial diseases have very different mechanisms (inflammatory versus mechanical) but both may have a severe impact on patients’ health related quality of life (HRQoL) in terms of physical, mental and social well-being though these have not been directly compared [3–13]. There are differences in treatment options between these diseases: on top of non-inflammatory anti-steroidal drugs and physiotherapy, biologic treatments such as anti-tumor necrosis factors have been proven to be successful in reducing AxSpA symptoms and in improving HRQoL [14], whereas for low back pain, analgesics and active physiotherapy are recommended.

The dispositional and global expectations individuals hold about success or failure are described by the constructs of optimism and pessimism. Dispositional optimism is defined as a stable, trait-like personality characteristic consisting of a general positive mood or attitude about the future with a tendency to expect favorable outcomes in life situations [15]. In opposition to dispositional optimism, dispositional pessimism is the tendency to generally expect negative outcomes in the future [15].

In different medical settings, optimistic people have been shown to have higher QoL compared to people with low optimism levels or pessimistic people [15–17]. This has been investigated in particular in people with cancer, epilepsy, haemodialysis and in patients having undergone aortic-coronary bypass [18–21]. In chronic rheumatic diseases, the few studies available also indicate optimism seems positively correlated to HRQoL [22–24]. However, none of these studies was focused on axial diseases.

Exploring the relationship between optimism and HRQoL in AxSpA and chronic low back pain could have interesting practical implications. Indeed, as research has linked optimism to lower pain sensitivity and better adjustment to chronic pain [25], it could provide important insights for clinicians regarding treatment decisions in particular in AxSpA where pharmacological treatment with biologic agents is decided mainly based on the patients’ subjective perception of their own disease [26]. Moreover, low back pain is a complex multifactorial process influenced by somatic, psychological and environmental factors, and falls within the biopsychosocial model of disability and health [27, 28]. Therefore, investigating levels of optimism among chronic low back pain patients can bring insights for targeting treatment such as coping enhancement techniques. Given the differences between AxSpA and low back pain, in terms of physiopathology, prognosis and treatment options, it could be expected that optimism levels might be different, and that the links between optimism and HRQoL might be different [25].

Thus, the objectives of the present study were to explore the levels of HRQoL and optimism in AxSpA and low back pain, and the relationship between optimism and HRQoL in these two populations.

## Methods

### Design

This study was a multi-centre cross-sectional study from the rheumatology departments of the Pitié-Salpetrière hospital in Paris and the René Dubos hospital in Pontoise as well as from two outpatient clinics from private practice rheumatologists in Paris, France. This study was approved by the Pitié-Salpetrière Ethic committee.

### Participants and procedure

All AxSpA patients according to the internationally recognised Assessment of Spondyloarthritis (ASAS) criteria [29] seen in hospitalization or outpatients visits in the centers between September 2013 and February 2014 were contacted by mail and asked to fill-out a self-report questionnaire. Furthermore, patients diagnosed with chronic low back pain (pain persisting more than 3 months) or subacute low back pain (pain lasting between 4 to 12 weeks) considered mechanical by the physician according to European guidelines for the management of low back pain, and seen between October 2013 and February 2014 were included in the study [30, 31]. All patients were 18 years-old or more, spoke French, signed an informed consent and were able and willing to fill out a questionnaire.

### Data collection

Demographic characteristics including age, gender, body mass index, education level, family structure (marital status), and work status (employed, unemployed or unable to work) were collected. The medical files were accessed for each patient to confirm the diagnosis [29], and for AxSpA patients, to quantify disease activity and severity.

Optimism was assessed by the French version of the Life Orientation Test-Revised (LOT-R) [32, 33]. The LOT-R consists of 10 questions with a 6-item measure and 4 filler items assessing individual differences in generalized optimism versus pessimism. The total score ranges from 0 to 24 with high scores indicating higher levels of optimism: for indicative purposes we analysed optimism using cutoff values as follows: 0–13 indicates low optimism, 14–18 moderate optimism and 19–24 high optimism. Sub-scales of optimism and pessimism are useful both when compared against one another and combined for practical use in a clinical setting [34].

HRQoL was measured by the French version of the Short Form Health Survey SF-12, a self-administered, generic health related quality of life (HRQoL) instrument assessing function and well-being via multi-item scales measuring 8 domains: physical function, role physical, role emotional, vitality, mental health, social functioning, general health and bodily pain [35]. The SF12 has two subscores, a physical composite score (PCS) and a mental composite score (MCS).

For all patients, anxiety and depression were assessed by the Hospital Anxiety and Depression Scale (HADS) [36] and Visual Analogue Scales were used to assess pain and global assessment. For AxSpA patients only, disease activity was measured by the Bath Ankylosing Spondylitis Disease Activity (BASDAI), a widely used subjective measure consisting of 6 questions rated from 1 (no problem) to 10 (worst problem) assessing various aspects of the disease such as pain, fatigue and morning stiffness [37, 38]. The functional activity was assessed by the Bath Ankylosing Spondylitis Functional Index (BASFI), a 10-item measure rated on a 1 (easy) to 100 (impossible) scale assessing function in daily life activities [39].

### Statistical analysis

To evaluate the relationship between optimism and HRQoL in the two populations, Pearson correlations were performed. Multivariable linear regression analyses were performed to explain HRQoL (PCS or MCS) by optimism, taking into account: demographic variables, marital and work status, global assessment VAS, duration of symptoms, anxiety, depression, and BASFI and BASDAI for AxSpA patients. Only patients with available LOT-R and SF12 were included in the analysis (no imputation of missing data). The R statistical software version 3.0.2 was used; a *p* value <0.05 was considered significant.

## Results

### Study population

Initially, 462 patients (321 AxSpA and 131 low back pain) were contacted; 307 (64 %) answered; and 288 (62 %) patients had analysable data: 199 (69 %) with AxSpA and 89 (31 %) with low back pain.

The mean age of the 288 patients was 47.3 years (standard deviation (SD) 11.9, range 19–85), 140 (48.6 %) were men, and the majority were married (*N* = 198, 68.8 %), had a higher education level (*N* = 175, 61.2 %) and were currently employed (*N* = 165, 61.8 %) (Table 1). The AxSpA patients carried the HLA B27 genotype (*N* = 116, 58.9 %), their radiographs or magnetic resonance imaging often showed sacroiliitis, the hallmark of this disease (*N* = 119, 61 %) and their disease was moderately active and severe: BASDAI 3.8 (2.0), BASFI 28.3 (25.7).

**Table 1:** Patient characteristics for199 AxSpA patients and 89 LBP patients

Variables	All	AxSpA	LBP	*p*-value
	N = 288	N = 199	N = 89	
Age, years, mean (SD)	47.3 (11.9)	45.9 (11.4)	50.5 (12.5)	**0.0028**
Male gender, N (%)	140.0 (48.6)	98.0 (49.3)	42.0 (47.2)	0.75
BMI, kg/m^2^, mean (SD)	25.9 (11.7)	25.3 (13.7)	26.0 (5.2)	0.92
Higher education, N (%)	175 (61.2)	124 (62.3)	51.0 (57.3)	0.78
Married or couple, N (%)	198.0 (68.8)	141 (70.8)	57.0 (65.0)	0.30
Current paid employment, N (%)	165 (61.8)	118 (63.1)	47 (58.8)	0.76
Pain VAS, mean (SD)	4.4 (2.4)	4.5 (2.4)	4.3 (2.4)	0.45
Global assessment VAS, mean (SD)	4.5 (2.3)	4.5 (2.4)	4.6 (2.2)	0.97
Duration of symptoms, years mean (SD)	14.4 (11.2)	15.2 (10.8)	12.5 (12.2)	0.07
HADS Anxiety, mean (SD)	8.3 (3.9)	8.3 (3.9)	8.5 (3.9)	0.61
HADS Definite Anxiety, N (%)	78 (27.4)	55 (27.8)	23 (26.4)	0.70
HADS Depression, mean (SD)	5.7 (3.7)	5.6 (3.6)	6.0 (3.8)	0.31
HADS Definite Depression, N (%)	35 (12.8)	23 (12.3)	12 (13.8)	0.91
SF12 PCS, mean (SD)	43.12 (7.9)	43.7 (8.2)	41.9 (7.1)	0.07
SF12 MCS, mean (SD)	46.2 (8.0)	45.9 (7.8)	46.7 (8.1)	0.32
Optimism, LOT-R, mean (SD)	13.8 (4.2)	13.8 (4.2)	13.9 (4.0)	0.78
High optimism (LOT-R >19) N (%)	35 (12.2)	24 (12.1)	11 (12.4)	0.62
Low Optimism (LOT-R <13) N (%)	126 (45.5)	82 (43.6)	44 (49.4)	

Results are reported on available data. Missing data were rare (<5 %)

Significant *p* values are represented in bold characters

*SD* Standard Deviation, *BMI* Body Mass Index, *VAS* Visual Analogical Scale, range 0-10, *HADS* Hospital Anxiety and Depression Scale, *SF12 PCS* Short Form 12, Physical Composite Score, *SF12 MCS* Short Form 12, Mental Composite Score, *LOT-R* Life Orientation Test-Revised

Higher education: post high school éducation

### HRQoL and optimism levels

No significant differences were found between levels of HRQoL and levels of optimism between the two populations (Table 1 and Additional file 1: Figure S1): optimism was in the moderate range: 13.8 ± 4.2 and HRQoL was low (Table 1).

### Relation between optimism and HRQoL

For AxSpA patients, optimism was positively correlated to the mental component of HRQoL only (rho = 0.54, *p* <0.01) whereas it was not correlated to the physical component (rho = 0.19, *p* = 0.07). For low back pain patients however, optimism was positively correlated to both component of HRQoL with a stronger correlation for the mental component (MCS: rho = 0.58, *p* <0.01; PCS: rho = 0.26, *p* <0.01).

Multivariable linear regression (Table 2) indicated that optimism was related to MCS in both populations (AxSpA: ß = 1.03, *p* <0.0001; low back pain: ß = 1.40, *p* <0.0001). However, optimism was not related to PCS; rather, the elements associated with PCS were global assessment visual analogue scale and body mass index in both populations; and work status, BASDAI and BASFI in AxSpA (Table 2). The other variables analysed were not significant.

**Table 2:** Multivariable linear regression analysis to explain HRQoL: PCS and MCS

	AxSpA				LBP			
	PCS		MCS		PCS		MCS	
	Beta		Beta	*p* value	Beta	*p* value	Beta	*p* value
Optimism, LOT-R	−0.045	0.68	1.03	**<0.0001**	0.18	0.39	1.40	**<0.0001**
BMI, for an increase of 3 points	−052	**0.022**	−0.37	0.159	−1.63	**<0.001**	0.60	0.3367
Work status (paid employment)	2.06	**0.021**	−0.63	0.54	−1.37	0.30	−0.52	0.78
Global assessment VAS	−1.1	**<0.0001**	−0.52	0.08	−1.48	**<0.001**	−0.76	**0.037**
BASDAI	−0.79	**0.014**	−1.2	**0.0026**	N/A	N/A	N/A	N/A
BASFI	−0.10	**0.0001**	−0.05	0.30	N/A	N/A	N/A	N/A

Significant *p* values are represented in bold characters

*BMI* Body Mass Index; *VAS* Visual Analogical Scale; *BASDAI* Bath Ankylosing Spondylitis Disease Activity; *BASFI* Bath Ankylosing Spondylitis Functional Index

## Discussion

This study shows AxSpA and low back pain patients had a decreased HRQoL, interestingly very similar in both diseases. Levels of optimism were lower than in the general population [38] and were similar in both diseases, in the moderate range. Furthermore, there was a positive relation between optimism and mental HRQoL, but not physical HRQoL.

The present study has strengths and limitations. The issues for external validity include that the sample size was relatively small and all patients came from Paris, France; however representativity was increased by recruitment from both tertiary care and private practices and the patient characteristics are in keeping with usual data for these populations. Furthermore optimism levels have been shown to be consistent across countries [40]. Sample sizes were different for both diseases and rather low for low back pain; this is due to the sampling method (convenience sample); thus low back pain results should be interpreted with caution. Moreover, there is a lack of data on optimism levels in the general population, and on cutoff values to interpret the LOT-R even though the LOT-R is a widely used questionnaire to assess optimism [18–21, 32]. Pessimism was not assessed in this study.

In this study, AxSpA patients as well as low back pain patients suffered from poor HRQoL in terms of physical and mental well-being. Although there was no control group in this study, the use of SF12 (for which the results are calibrated in the general population) allows an assessment of HRQoL compared to the general population [41].

These findings are in line with previous studies using the longer version of the SF-12, the SF-36 [3–12, 42]. In 2 Spanish studies using the SF12, HRQoL results differed from the present findings; in AxSpA, patients had lower PCS and higher MCS [43] and in low back pain, patients had lower PCS and MCS [44]. The differences observed may be due to sampling differences. Indeed, regarding low back pain patients, there was no indication about disease duration and low back pain was self-assessed. This could explain why these patients suffer from a poorer HRQoL. Regarding AxSpA patients, they reported higher disease activity and lower functionality, which could explain the lower scores on the physical component of the SF-12.

The present study indicated HRQoL was similar in both diseases. This is an interesting finding, given the different physiopathological nature of these diseases. It indicates the physical impact of these diseases may be close, perhaps due to similar functional limitations because of the spinal involvement. Regarding mental health, we anticipated that low back pain patients would have a lower mental HRQoL than AxSpA patients due to the lack of treatments and the difficulty in reducing symptoms but this was not the case.

In this study, optimism could be considered for indicative purposes to be in the moderate range. Interestingly, optimism levels were similar in the 2 diseases studied and this may suggest that people suffering from either AxSpA or low back pain are rather less optimistic than a healthy population. Indeed, a study assessing optimism among 504 high school students reports a mean LOT-R of 16.5 whereas in this study, the mean LOT-R of the whole sample was 13.8 [45]. Even though dispositional optimism is considered as a relatively stable feature of the personality over time and context, there are variations in optimism when people are prepared to face a threat and this indicates that for some people, optimism levels may vary [46–48].

Optimism was related to mental HRQoL both in univariate and multivariate analyses in the present study. The link between moderate optimism and low quality of life might be explained by the role of coping strategies used by the patients. Indeed, it could be that less optimistic people use more dysfunctional coping strategies when confronting stressful events than optimistic people [49–53].

The present findings were consistent with the previously published studies on optimism in the field of rheumatic diseases and chronic conditions [22–24]. The largest study investigating the relationship between optimism and HRQoL in 1529 patients with chronic diseases found that optimism was positively linked with HRQoL [23]. Similarly, Tsakogia and colleagues study [22] investigated the influence of optimism on the HRQoL of patients with musculoskeletal problems and found that dispositional optimism was an independent factor affecting the mental composite score of the SF-12, and weakly (similarly to the present findings), the physical component of SF12. However, in comparison to these two studies, the mean LOT-R of our sample appeared lower.

In the present study, optimism had a very small effect on the physical HRQoL. These results indicate that optimism may not influence the interpretation of physical scores, widely used in the evaluation of AxSpA and low back pain.

The present study evidenced non-psychological drivers of PCS including global assessment for both AxSpA and low back pain patients, and BASFI for AxSpA patients only. These results confirm the validity of the study.

Applying the bio-psychosocial model to chronic diseases implies taking into account not only disease activity and severity, but also psychological aspects including optimism. We have shown optimism is a driver of psychological, but not physical, HRQoL in the 2 chronic diseases. However, further studies are needed to understand the pathway linking optimism to HRQoL and the potential factors playing a role in this relationship such as coping techniques.

## Additional file

Additional file 1: Figure S1. LOT-R in 288 patients.
