# Supplementary figures and images for: Relationship between optimism and quality of life in patients with two chronic rheumatic diseases: axial spondyloarthritis and chronic low back pain: a cross sectional study of 288 patients
Source: Health Qual Life Outcomes. 2015 Jun 10;13:78. doi: 10.1186/s12955-015-0268-7 (PMC4491882; doi:10.1186/s12955-015-0268-7)

**Figure S1**: LOT-R in 288 patients


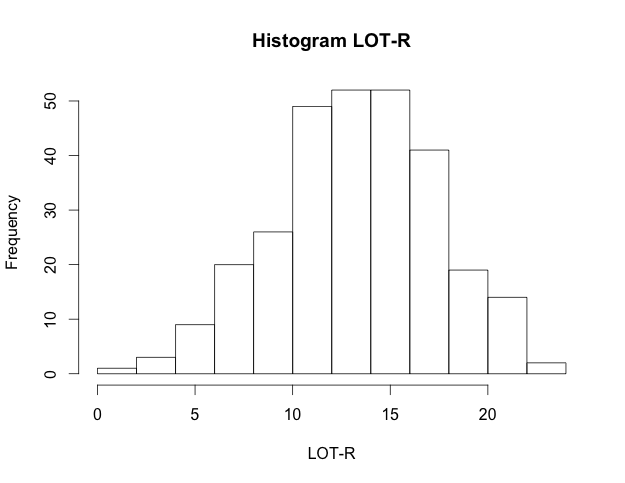

Supplement: Additional file 1: Figure S1. — LOT-R in 288 patients. [file 12955_2015_268_MOESM1_ESM.doc]
